# Enumeration of Viable Non-Culturable *Vibrio cholerae* Using Droplet Digital PCR Combined With Propidium Monoazide Treatment

**DOI:** 10.3389/fcimb.2021.753078

**Published:** 2021-11-02

**Authors:** Shuo Zhao, Jingyun Zhang, Zhe Li, Yu Han, Biao Kan

**Affiliations:** State Key Laboratory of Infectious Disease Prevention and Control, National Institute for Communicable Disease Control and Prevention, Chinese Center for Disease Control and Prevention, Beijing, China

**Keywords:** viable but non-cultivable state, ddPCR, qPCR, viable cell counting, propidium monoazide, *Vibrio cholerae*

## Abstract

Many bacterial species, including *Vibrio cholerae* (the pathogen that causes cholera), enter a physiologically viable but non-culturable (VBNC) state at low temperature or in conditions of low nutrition; this is a survival strategy to resist environmental stress. Identification, detection, and differentiation of VBNC cells and nonviable cells are essential for both microbiological study and disease surveillance/control. Enumeration of VBNC cells requires an accurate method. Traditional counting methods do not allow quantification of VBNC cells because they are not culturable. Morphology-based counting cannot distinguish between live and dead cells. A bacterial cell possesses one copy of the chromosome. Hence, counting single-copy genes on the chromosome is a suitable approach to count bacterial cells. In this study, we developed quantitative PCR-based methods, including real-time quantitative PCR (qPCR) and droplet digital PCR (ddPCR), to enumerate VBNC *V. cholerae* cells by counting the numbers of single-copy genes in samples during VBNC-state development. Propidium monoazide (PMA) treatment was incorporated to distinguish dead cells from viable cells. Both PCR methods could be used to quantify the number of DNA copies/mL and determine the proportion of dead cells (when PMA was used). The methods produced comparable counts using three single-copy genes (VC1376, *thyA*, and *recA*). However, ddPCR showed greater accuracy and sensitivity than qPCR. ddPCR also allows direct counting without the need to establish a standard curve. Our study develops a PMA-ddPCR method as a new tool to quantify VBNC cells of *V. cholerae*. The method can be extended to other bacterial species.

## Introduction

Toxigenic *Vibrio cholerae*, a part of autochthonous aquatic ecosystems, is a reservoir for human infections and a threat to public health globally (Islam et al., 1994; Kaper et al., 1995; Harris et al., 2012). Previous research has shown that *V. cholerae* can enter into a dormant state, also called the viable but non-culturable (VBNC) state (Xu et al., 1982; Oliver, 2010). The first systematic study of the VBNC state in *V. cholerae* serogroup O1 was reported by Xu et al. in 1982 (Xu et al., 1982). This state was defined as metabolically active but lacking the ability to reproduce on routine culture media (Oliver, 2005). When exposed to an unfavorable environment (i.e., starvation, low temperature, sub-optimal redox conditions, irradiation and antibiotic pressure), many species of bacteria can enter a VBNC state for long-term survival (Ducret et al., 2014; Pinto et al., 2015; Wu et al., 2016; Zhang et al., 2021). In the laboratory setting, the VBNC state of *V. cholerae* is usually induced by incubation in artificial sea water (ASW) at 4°C (Asakura et al., 2007).

Differentiation between *V. cholerae* in the VBNC state and dead cells is challenging, especially in samples from aquatic ecosystems where the cell concentration is low (Davis, 2014). Propidium iodide staining can be combined with the evaluation of cellular integrity by using an electronically programmable single-cell sorter (Caron et al., 1998). Several studies have used Live/Dead stains combined with microscopic examination or flow cytometry to identify VBNC cells (Nebe-von-Caron et al., 2000; Gonzalez-Escalona et al., 2006). However, FCM intermediate state results were poorly characterized and mainly rely on the experience of the operator (Strauber and Muller, 2010; Dong et al., 2020). These methods are limited in use.

Quantitative real-time PCR and real-time LAMP (qLAMP) combined with propidium monoazide treatment (PMA-qPCR and PMA-qLAMP) are methods to detect VBNC cells (Nocker et al., 2006; Cao et al., 2019). PMA can penetrate damaged cell membranes. Intercalation of DNA by PMA inhibits PCR amplification. Thus, PMA-qPCR has become a popular method for distinguishing between live and dead bacteria (Banihashemi et al., 2012; Wu et al., 2015). In our previous study, we found that treatment of *V. cholerae* with 20 μM PMA for 20 min was optimal (Wu et al., 2015).

There are extensive guidelines for the selection of reference genes for new qPCR experiments, and preliminary experiments are very important (Bustin et al., 2009). Nevertheless, most qPCR analyses are carried out using only one reference gene. Moreover, PCR efficiency and analytical parameters are frequently omitted from literature reports. A single-copy gene is a housekeeping gene with only one copy in the genome. Averaging data for three single-copy genes is recommended for qPCR analyses.

The droplet digital polymerase chain reaction (ddPCR) is a unique method for quantifying the absolute copy number of a target gene without external criteria (Sun and Joyce, 2017; Li et al., 2018), i.e., ddPCR does not require a standard curve for quantification. In ddPCR, DNA samples are divided into large water-in-oil droplets and then amplified by PCR. Droplets are then individually analyzed by a fluorescence detector, enabling quantification of the target DNA. This method has higher sensitivity than qPCR (Sun and Joyce, 2017; Link-Lenczowska et al., 2018), and ddPCR is superior to qPCR in accuracy in detecting plasmid-borne genes (Xu et al., 2021). ddPCR data correlates well with qPCR data (Li et al., 2014; Gobert et al., 2018). Analysis of stably-expressed single-copy genes by ddPCR may produce high-quality data, even when a low number of replicates is assayed or the sample is VBNC cells. PMA treatment combined with ddPCR analysis (PMA-ddPCR) has been adopted for quantification of bacterial survival in fecal samples (Gobert et al., 2018).

In this work, we developed a method based on PMA-ddPCR to quantify VBNC cells using VC1376 (encoding GGDEF family protein), *thyA* (encoding thymidylate synthase), and *recA* (encoding ATP-binding protein Uup), which are all single-copy genes in *V. cholerae*. We compared the data from ddPCR with that obtained by qPCR. Our results show that ddPCR of single-copy genes combined with PMA treatment is a practical and effective approach for counting VBNC *V. cholerae* cells.

## Materials and Methods

### Bacterial Strain and Culture Conditions

*V. cholerae* O1 El Tor strain C6706 is toxigenic, which is preserved in our laboratory. We performed the experiments with C6706 in our biosafety level 2 (BSL-2) laboratory with the corresponding biosafety protection measures. This strain was initially stored in 20% (v/v) glycerol at −80°C, then cultured on nutrient agar. Three single colonies of the strain were picked and suspended in Luria-Bertani (LB) broth (Oxoid, Basingstoke, UK) and cultured overnight at 37°C with shaking (200 rpm). The cultures were then diluted into fresh LB broth (1:50, v/v), incubated at 37°C with shaking at 200 rpm, and grown to mid-exponential phase. The cultures were then washed twice with ASW and diluted to OD_600_ = 1.0 [approximately 1×10^9^ colony-forming units (CFU)/mL]. Finally, the cultures were inoculated into ASW at a final concentration of 1.3×10^7^ CFU/mL. ASW was prepared using sea salt (40 g/L; Sigma-Aldrich, Inc., St. Louis, MO, USA) and was sterilized using 0.22-μm membrane filters (Millipore, USA). ASW was used for the culture of *V. cholerae* to simulate the natural environment of *V. cholerae*, to study the VBNC state development of it. LB medium is prepared according to the common formula, for the laboratory culture of *V. cholerae*.

### Induction of VBNC State

Exponential-phase cells were kept in ASW at 4°C with oxygen limitation, achieved by filling 2-mL vials to the brim with culture and closing the lid to exclude air. Three vials for each target gene as parallel were used for detection *V. cholerae*. Fresh vial was used for enumeration, and then that culture was discarded to prevent aeration.

### PMA Treatment

As described in our previous study (Wu et al., 2015), viable cells were enumerated using PMA (Biotium, USA) combined with qPCR or ddPCR. Briefly, 200-μL aliquots of cells kept at 4°C were treated with 20 mM PMA for 20 min in the dark, then exposed to light on ice for 15 min using a 650-W double-ended halogen lamp.

### DNA Isolation

DNA was isolated from 200-μL cell suspensions treated or not treated with PMA using a Wizard Genomic DNA Purification Kit (Promega, Madison, WI, USA) according to the manufacturer’s instructions. To obtain the maximum yield of DNA, a double elution was performed, using 200 μL of elution buffer each time. Purified DNA was quantified using a NanoDrop 2000c spectrophotometer (Thermo Scientific, Asheville, NC, USA). DNA samples were stored at −20°C.

### qPCR Analysis and Standard Curve

The mixture for qPCR amplification contained 1 μL of template DNA, Premix Ex *Taq* (TaKaRa, Dalian, China), 0.25 μM of each forward and reverse primer targeting the single-copy gene VC1376, *thyA*, or *recA* of *V. cholerae* O1 (**Table 1**), and ultrapure water to a final volume of 20 μL. qPCR was performed in a LightCycler 96 system (Roche, Indianapolis, IN, USA). All qPCR reactions were performed in triplicate. The thermal cycling conditions were: 3 min at 95°C; followed by 40 cycles of 5 s at 95°C and 30 s at 50°C for VC1376, 54°C for *thyA*, and 55°C for *recA*; then melt curve analysis from 65°C to 95°C with increments of 0.5°C for 5 s each. The enumeration results were considered negative if the Cq value was >36.

**Table 1:** Oligonucleotides used in this study.

Primer	Sequence (5’-3’)	References
VC1376-F	TAACATAATAAGGAAGAAGTGGAT	Wu et al., 2015
VC1376-R	ACAGTCAGAAGCAGAGAA	
thyA-F	ACATGGGACGCGTGTATGG	This study
thyA-R	ATATGACCACCATCAGGCTTAGC	
RecA-F	GTGCTGTGGATGTCATCGTTGTTG	Kotetishvili et al., 2003
RecA-R	CCACCACTTCTTCGCCTTCTTTGA	

To prepare standard curves, DNA was extracted from purified *V. cholerae* O1 El Tor strain C6706, and the standard curves were made by serially diluting this DNA.

### ddPCR Analysis

EvaGreen^®^ chemistry-based ddPCR and data analysis was performed according to the manufacturer’s instructions; EvaGreen Supermix (2×) was purchased from Bio-Rad Laboratories. PCR was performed in a 20-µL volume containing 10 mL 2× EvaGreen Supermix, 1 mL DNA, 0.2 µM of each primer, and 8.8 µL ddH_2_O. For negative controls, 5 µL water were added instead of genomic DNA. Each sample was quantified twice. A Bio-Rad Automated Droplet Generator was used to generate droplets. Thermal cycling was performed using a Bio-Rad C1000 Touch™ Thermal Cycler, with conditions: 95°C for 5 min; 40 cycles of 30 s at 95°C and 30 s at 50°C for VC1376, 54°C for *thyA*, and 55°C for *recA*; and a final incubation 4°C for 5 min and 90°C for 5 min. After reaction, droplets were analyzed using a QX200™ Droplet reader. Data analysis was done using Bio-Rad QuantaSoft™ software.

### Live/Dead Staining

Live/Dead staining was performed as described previously (Almagro-Moreno et al., 2015). We centrifuged 1 mL aliquots from each sample at 2152 × *g* for 2 min, and the pellet was resuspended in 1 mL phosphate-buffered saline. The cells were then stained with a 3-µL mixture of SYTO9 and propidium iodide (1:1 v:v; Molecular Probes, Eugene, OR, USA) per 1 mL of suspension. After incubation in the dark for 15 min at 25°C, the stained cells were mounted on a glass slide and low-fluorescence immersion oil was added on the coverslip. The cells were then examined with a Nikon ECLIPES 80i microscope. Images were captured with NIS-Elements F3.2 microscopy software (Nikon).

### Statistical Analysis

Figures were drawn using GraphPad Prism software from three replicate values. Each replicate value was the mean value from a triplet measurement.

## Results

### Sensitivity of qPCR and ddPCR Quantification

First, we determined the sensitivity of the qPCR and ddPCR assays for *V. cholerae* genes. The average concentration of the *V. cholerae* strain C6706 cells used (culture at 37°C with shaking at 200 rpm, to mid-exponential phase) was around 1.3×10^7^ CFU/mL, as determined by plate counting. Chromosomal DNA extracted from *V. cholerae* strain C6706 was used as the template for amplification, at 10 ng/µL to 1 fg/µL (prepared by serial 10-fold dilution).

Standard curves for quantification of the target genes VC1376, *thyA*, and *recA* by qPCR were constructed in the range 1.3×10^1^ to 1.3×10^5^ copies/µL of C6706 DNA. The slopes were −3.26, −3.23, and −3.34 for VC1376, *thyA*, and *recA*, respectively. The mean qPCR efficiencies for VC1376, *thyA*, and *recA* were 99%, 100%, and 103%, respectively. The lower limit of detection using SYBR Green (tested in triplicate) was 10 fg/µL of total DNA, with mean Cq values of 35.58 ± 0.24, 35.02 ± 0.96, and 34.06 ± 0.61 for VC1376, *thyA*, and *recA*, respectively (**Table 2**).

**Table 2:** Gene quantification (VC1376, *thyA*, and *recA*) by qPCR and ddPCR from a 10-fold serial dilution of genomic DNA of *V. cholerae* strain C6706.

qPCR						
Concentration of Template	VC1376		*thyA*		*recA*	
	Cq mean	SD	Cq mean	SD	Cq mean	SD
10 ng/μL	16.11	0.08	16.73	0.16	14.17	0.11
100 pg/μL	23.21	0.08	23.48	0.17	21.27	0.12
10 pg/μL	26.93	0.10	27.18	0.19	25.01	0.18
1 pg/μL	29.71	0.15	30.25	0.21	27.90	0.48
100 fg/μL	32.64	0.45	32.33	0.86	31.52	0.64
10 fg/μL	35.58	0.24	35.02	0.96	34.06	0.61
1 fg/μL	UD	UD	UD	UD	UD	UD
R^2^	0.997		0.995		0.999	
ddPCR						
Concentration of Template	VC1376		*thyA*		*recA*	
	copies/μL*	SD	copies/μL*	SD	copies/μL*	SD
10 ng/μL	ULOD	ULOD	ULOD	ULOD	ULOD	ULOD
100 pg/μL	913.00	18.38	1253.00	16.97	711.50	19.09
10 pg/μL	118.00	7.07	119.50	9.19	64.30	12.30
1 pg/μL	10.70	0.71	11.45	0.92	8.65	0.49
100 fg/μL	1.20	0.14	1.60	0.42	1.45	0.21
10 fg/μL	0.17	0.03	0.22	0.02	0.27	0.02
1 fg/μL	0.03	0.02	0.07	0.01	0.08	0.02
R^2^	0.997		0.992		0.999	

*The amount of DNA in each reaction.

UD, undetected. ULOD, DNA concentration at which the signal of the assay was saturated (>20,000 copies in the reaction mixture).

To determine the lower limit of detection by ddPCR, six replicates were performed with the three single-copy genes used to construct the qPCR standard curves. High linearity was observed. With 10 fg/µL of total DNA, a mean of 0.17 ± 0.03, 0.22 ± 0.02, and 0.27 ± 0.02 copies/µL of VC1376, thyA, and recA were detected, respectively. Moreover, the detection sensitivity reached ≤1 fg of total DNA (0.03 ± 0.02, 0.07 ± 0.01, and 0.08 ± 0.02 copies/µL, respectively) (**Table 2**). Therefore, ddPCR was 10 times more sensitive than qPCR. Fluorescence-labeled curve reaction saturation of ddPCR was reached at 100 pg/µL (i.e., >20,000 positive droplets).

Both PCR methods showed good linearity in the range of quantification, with R^2^ values between 0.992 and 0.999 for the three genes. The number of DNA copies/µL in samples not treated with PMA determined using genes VC1376, thyA, and recA was similar using either qPCR or ddPCR.

### Enumeration of VBNC Cells by qPCR and ddPCR

**Figure 1:**
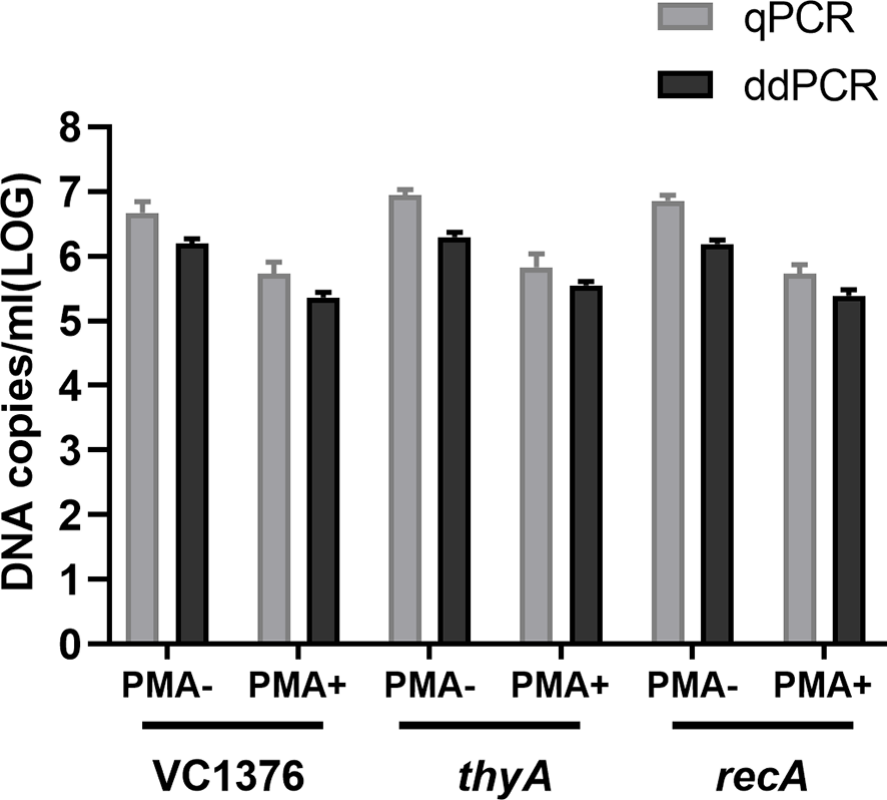
Quantification of total and viable cells (in DNA copies/mL) using genes VC1376, *thyA*, and *recA* at day 70 during viable but non-culturable (VBNC)-state development of *Vibrio cholerae* strain C6706. Total cells were measured without PMA treatment (PMA−) and viable cells were measured after PMA treatment (PMA+). qPCR, quantitative PCR; ddPCR, droplet digital PCR. Error bars represent the mean and standard error of measurement.

Both qPCR and ddPCR methods were used to quantify DNA copies from VBNC-state cells; samples were taken on day 70 of the VBNC-state development of *V. cholerae* strain C6706. All three genes (VC1376, thyA, and recA) were used as targets for enumeration. Total VBNC cells and VBNC-state living cells were enumerated by PCR without or with PMA treatment (**Figure 1**). We report the enumeration of cells per mL in terms of the number of DNA copies of the three genes per mL (noting that there is one copy of each gene per cell).

Similar numbers of total cells (i.e., live + dead) were determined by ddPCR using VC1376 and recA (6.18 ± 0.10 and 6.20 ± 0.19 log_10_ DNA copies/mL, respectively), and slightly higher values were determined using thyA (6.29 ± 0.13 log_10_ DNA copies/mL). Quantification of viable cells by ddPCR gave values 5.4 ± 0.06 and 5.39 ± 0.16 log_10_ DNA copies/mL using VC1376 and thyA respectively; a slightly higher value was determined using recA (5.55 ± 0.10 log_10_ DNA copies/mL).

The loss of cellular viability was evaluated from the cell counts determined by qPCR or ddPCR and the counts determined by PMA-qPCR or PMA-ddPCR, respectively. Using qPCR, the viability loss determined using VC1376, thyA, and recA was 0.7, 0.58, and 1.13 log_10_ DNA copies/mL, respectively. Using ddPCR, the viability loss determined using VC1376, thyA, and recA was 0.78, 0.74, and 0.81 log_10_ DNA copies/mL, respectively.

The consistency of the ddPCR-derived data was better than that obtained by qPCR when tested with samples from day 70 of VBNC-state development (**Table 3**). Here we calculated the proportion of VBNC-state cells using the absolute value of live cells (DNA copies/mL in samples with PMA treatment) and the total number of DNA copies/mL (samples without PMA treatment). Quantification of the VBNC cell proportion by qPCR was 13.09%, 38.91%, and 9.30% when determined using VC1376, thyA, and recA, respectively, showing low consistency of the data produced using these test genes. However, when measured using ddPCR, the VBNC cell proportions were 16.09%, 18.06%, and 15.61% for VC1376, thyA, and recA, respectively.

**Table 3:** Assessment of viability loss rate of *V. cholerae* strain C6706 on day 70 of VBNC-state development by quantification of VC1376, *thyA*, and *recA* by qPCR and ddPCR.

Gene	Method	VBNC rate	Viability loss rate
VC1376	qPCR	13.09%	86.91%
	ddPCR	16.09%	83.91%
thyA	qPCR	38.91%	61.09%
	ddPCR	18.06%	81.94%
recA	qPCR	9.30%	90.7%
	ddPCR	15.61%	84.39%

### Quantification of Viable Cells During VBNC-State Development

**Figure 2:**
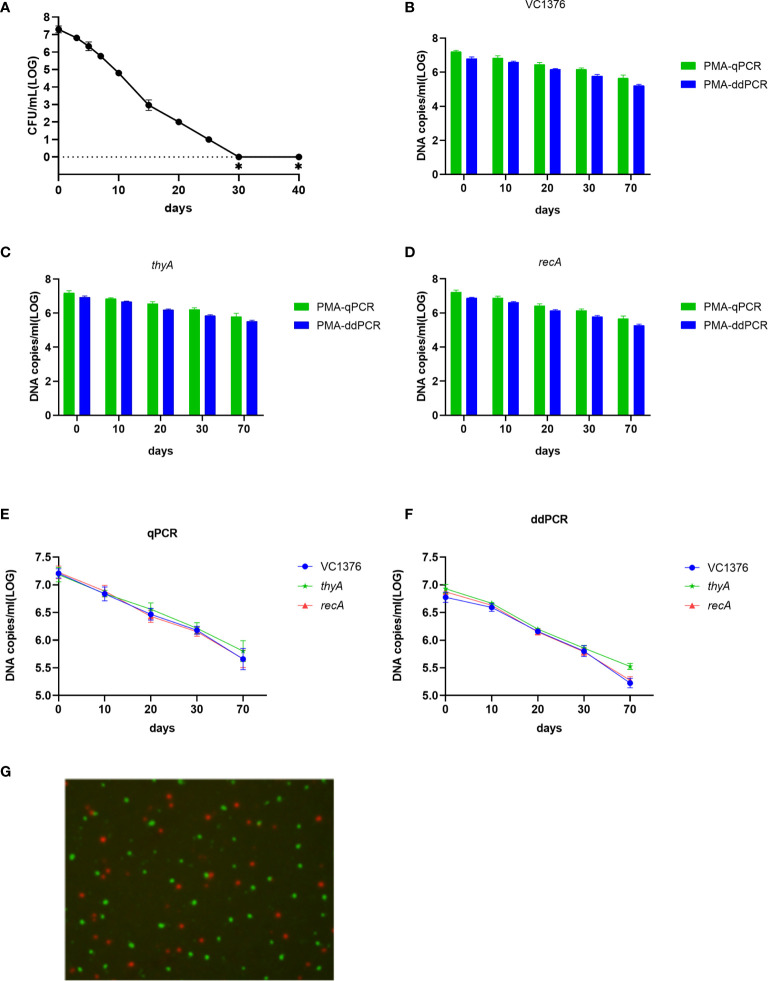
Enumeration of viable cells by qPCR and ddPCR during VBNC-state development of *V. cholerae* strain C6706. **(A)** Culturable cell counts during VBNC-state development of strain C6706. ∗ indicates that the culturable cell count was <1 colony-forming unit/mL. **(B–D)** DNA copies/mL measured using genes VC1376, *thyA* and *recA* after PMA treatment. **(E, F)** Change in number of DNA copies/mL measured by qPCR or ddPCR for determination of viable cell counts during VBNC-state development. **(G)** Live/Dead staining of cells of *V. cholerae* strain C6706 on day 70 of VBNC-state development. Viable cells, which are stained only by SYTO9, appear green; dead bacteria appear red.

Development of the VBNC state of *V. cholerae* is a process. Day 70 was the endpoint of observation of the VBNC state in our study. Culture samples with and without PMA treatment were collected on days 0, 10, 20, 30 and 70. Cellular DNA was extracted and determined by qPCR and ddPCR. The culturable cell counts (determined on LB-agar plates) decreased gradually and fell to zero after 30 days (**Figure 2A**). Viable cell counts were confirmed by Live/Dead staining, PMA-qPCR, and PMA-ddPCR (**Figure 2**). The viable cell counts determined by PMA-qPCR and PMA-ddPCR using VC1376, thyA, and recA showed a slow decrease from day 0 to day 70, from 6.09–7.01 log_10_ DNA copies/mL to 5.21–6.23 log_10_ DNA copies/mL (**Figures 2E, F**). Based on the data presented in Figuers 2B-F, a slightly lower count of DNA copies/mL was determined by PMA-ddPCR than by PMA-qPCR at each time point and for each gene, and the values obtained by qPCR had higher variance than the ddPCR data. Therefore, ddPCR had higher repeatability than qPCR in the counting of gene copies.

## Discussion

Development of the VBNC state of bacteria progresses with loss of culturability but preservation of viability (Trevors, 2011). Therefore, viable cell counts and culturable cell counts are the main indicators to reflect the progress of development of the VBNC state. Differentiation of VBNC-state *V. cholerae* from dead cells is challenging, especially in environmental samples (Davis, 2014). The reference methods used to identify the presence of *V. cholerae* are mostly culture-based; as VBNC cells cannot, by definition, be cultured, alternative methods must be developed to count VBNC cells. Methods based on cellular activity, the exploitation of physiological responsiveness (Kogure et al., 1979), and nucleic acid synthesis (Lleo et al., 2000) can be used to qualitatively detect viable cells. Each viable cell has an intact membrane and one copy of the chromosomal DNA, which may allow enumeration of VBNC cells by counting single-copy genes in the chromosomal DNA. In this study, we evaluated the use of PMA-ddPCR to quantify *V. cholerae* VBNC cells, and compared the data with that obtained by PMA-qPCR.

One major difference between qPCR and ddPCR quantification was the minimum positive copy number. While qPCR was not precise below 10 fg/µL of total DNA, the detection limit of ddPCR was 1 fg/µL of total DNA. This difference was of particular importance—ddPCR can be used to determine accurate results with a low number of VBNC cells. Thus, VBNC cells could be quantified in more samples (e.g., of environmental waters) by ddPCR. The inability to use qPCR to accurately quantify low numbers of VBNC cells has been reported previously (Lahteinen et al., 2014), and the greater capability of ddPCR to quantify small amounts of target molecules compared with qPCR is also in agreement with literature data (Gobert, et al., 2018; Xu et al., 2021).

In this study, we selected three single-copy genes to evaluate the counting of VBNC cells. One purpose of this was to use these genes for parallel verification of the quantitative PCR methods in gene copy counting; another was to compare results when testing with different genes using different primers and probes. Both qPCR and ddPCR, used in parallel, were successful for quantification of DNA copies in total and viable cells using VC1376, thyA, and recA as the target genes. The slight difference in the mean Cq values for the three genes probably resulted from different qPCR efficiencies of the primers, but the Cq values were similar, validating the method. However, the DNA copy numbers measured by qPCR had wider confidence intervals than those determined by ddPCR at each time point during development of the VBNC state, probably because ddPCR is an absolute counting method whereas qPCR relies on the amplification efficiency, Cq value, and calculation based on reference values. It was found that the DNA copy enumeration from the three genes by the two PCR methods was different (for example, the VBNC cell proportions and viability loss rates at day 70 of VBNC-state development were different), probably because of different amplification efficiencies. We found that quite similar gene copy enumeration was obtained when using genes VC1376, thyA, and recA, suggesting that these genes and the primers tested in this study can be used in the enumeration of VBNC cells.

This method has potential applications for identification the viable pathogens or other studies bacteria in samples, which cannot be conventionally cultured but still exist in VBNC state. Such as, it can be used in the distinguishment of dead and unculturable but alive bacteria in patient or food samples; or in the risk assessment of *V. cholerae* or other pathogens in the environment water, since the common culture methods cannot obtain the pathogen in VBNC state. In summary, we established and evaluated quantitative PCR-based methods to count viable but unculturable cells of *V. cholerae*, by counting single-copy chromosomal genes of VBNC-state cells. Treatment with PMA effectively excluded the chromosomal DNA of dead cells. ddPCR combined with PMA treatment was better for VBNC cell counting than qPCR, and can be used in VBNC-state research, and detection and surveillance of VBNC-state cells of *V. cholerae* in the environment. This approach could also be extended to other bacterial species.

## Data Availability Statement

The raw data supporting the conclusions of this article will be made available by the authors, without undue reservation.

## Author Contributions

BK conceived and designed the study. SZ, JZ, ZL, and YH contributed to the experiment. SZ and BK contributed to writing the manuscript. All authors contributed to the article and approved the submitted version.

## Funding

This work was supported by National Basic Research Priorities Program Grant 2015CB554201 and National Science and Technology Major Project 2018ZX10714002.

## Conflict of Interest

The authors declare that the research was conducted in the absence of any commercial or financial relationships that could be construed as a potential conflict of interest.

## Publisher’s Note

All claims expressed in this article are solely those of the authors and do not necessarily represent those of their affiliated organizations, or those of the publisher, the editors and the reviewers. Any product that may be evaluated in this article, or claim that may be made by its manufacturer, is not guaranteed or endorsed by the publisher.
